# High-Salt Intake Suppressed MicroRNA-133a Expression in Dahl SS Rat Myocardium

**DOI:** 10.3390/ijms150610794

**Published:** 2014-06-16

**Authors:** Tong-Shuai Guo, Jie Zhang, Jian-Jun Mu, Fu-Qiang Liu, Zu-Yi Yuan, Ke-Yu Ren, Dan Wang

**Affiliations:** 1 Department of Cardiovascular Medicine, First Affiliated Hospital of Medical College of Xi’an Jiaotong University, No. 277 Yanta West Road, Xi’an 710061, China; E-Mails: 215guotongshuai@163.com (T.-S.G.); Liufuqiang0909@163.com (F.-Q.L.); zuyiyuan@mail.xjtu.edu.cn (Z.-Y.Y.); renkeyu1008@163.com (K.-Y.R.); Dandelion622@gmail.com (D.W.); 2 Department of Cardiovascular Medicine, Xi’an No. 4 Hospital, No. 21 Jiefang Road, Xi’an 710004, China; E-Mail: zhangjie1988524@gmail.com

**Keywords:** salt-sensitivity, myocardial fibrosis, microRNA-133a

## Abstract

Salt-sensitive individuals show earlier and more serious cardiac damage than nonsalt-sensitive ones. Some studies have suggested that microRNA-133a could reduce cardiac hypertrophy and myocardial fibrosis. The current study aims to investigate the different functions of high-salt intake on salt-sensitive (SS) rats and Sprague-Dawley (SD) rats and the involvement of microRNA-133a in these roles. After high-salt intervention, the left ventricular mass (LVW) and left ventricular mass index (LVMI) of the salt-sensitive high salt (SHS) group were obviously higher than those of the salt-sensitive low salt (SLS) group. However, the difference between the Sprague-Dawley high salt (DHS) group and the Sprague-Dawley low salt (DLS) group was not significant. Compared with SLS group, collagen I and connective tissue growth factor (CTGF) in the heart of SHS group were significantly higher, whereas no statistical difference was observed between the DHS group and the DLS group. Compared with low-salt diet, microRNA-133a in the heart of both strains were significantly decreased, but that in the SHS group decreased more significantly. These results suggest that high salt intervention could down-regulate the expression of myocardial microRNA-133a, which may be one of the mechanisms involved in myocardial fibrosis in salt-sensitive hypertension.

## 1. Introduction

Hypertension is a primary risk factor for cardiovascular events, and salt-sensitivity is an intermediate genetic phenotype of essential hypertension. High salt intake can elevate blood pressure, and directly result in heart [1], brain [2] and kidney [3] damage independently of blood pressure. High salt intake promotes hyperplasia and hypertrophy of the myocardial cells and facilitates collagen deposition in myocardial cells, resulting in myocardial fibrosis and cardiac hypertrophy [4].

Over the years, much research attention has been directed toward microRNAs. MicorRNAs comprise a 22-nucleotide-long RNA sequence that can regulate protein synthesis by binding to the untranslated regions of targeted mRNAs, thereby altering stability of the target protein [5]. Studies have shown that microRNA-133a is mainly expressed in cardiac and skeletal muscles. It has several physiological and pathological effects, such as cardiac development [6], atthythmia [7], apoptosia [8], and smooth muscle differentiation [9]. Reports have indicated that microRNAs may be broadly involved in the pathological process of hypertensive cardiac hypertrophy and myocardial fibrosis. Care *et al.* [10] demonstrated that microRNA-133a is involved in cardiac hypertrophy. They identified three specific targets of microRNA-133a, namely, RhoA, Cdc42 and Nelf-A/WHSC2. All factors were involved in cardiac hypertrophy. Another study confirmed that microRNA-133 directly regulates the expression of transforming growth factor β (TGF-β) [11], connective tissue growth factor (CTGF) [12] and collagen I [13] in different models. However, very few reports have focused on whether high salt intake could regulate the expression of microRNA-133a and promote myocardial fibrosis.

The current study aims to observe the influence of high-salt intake on blood pressure, left ventricular weight, myocardial microRNA-133a and fibrosis levels by implementing high-salt diet intervention trial in Dahl salt-sensitive rats and SD rats. The final objective is to discuss the role of microRNA-133a in salt-sensitive hypertensive cardiac fibrosis with high-salt intervention.

## 2. Results

### 2.1. Effect of High-Salt Intake on Blood Pressure

**Figure 1:**
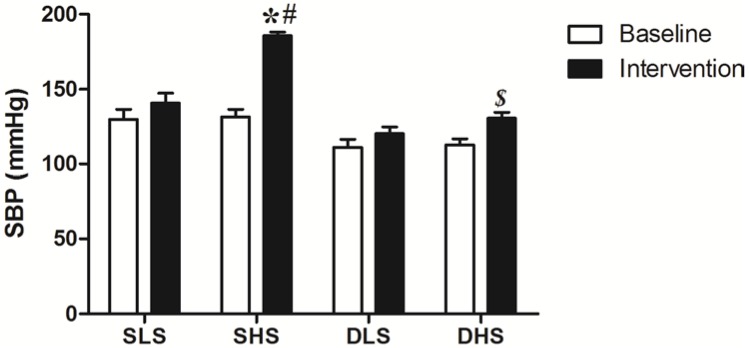
Systolic blood pressure (SBP) of rats before and after salt intervention. SLS (salt-sensitive low salt); SHS (salt-sensitive high salt); DLS (Sprague-Dawley low salt); DHS (Sprague-Dawley high salt). * *p* < 0.01 *vs.* SLS intervention; ^#^ *p* < 0.01 *vs.* DHS intervention; ***^$^*** *p* < 0.01 *vs.* DLS intervention.

At baseline, no statistical difference was observed in systolic blood pressure (SBP) in the same strain of rats (*p* > 0.05). After high-salt intake, SBP increased significantly in salt-sensitive (SS) rats and in Sprague-Dawley (SD) rats (SS: 185.5 ± 2.59 *vs.* 131.5 ± 5.01 mmHg, *p* < 0.01; SD: 130.5 ± 4.14 *vs.* 112.67 ± 4.03 mmHg, *p* <0.01). The increment of SBP in SS rats after high-salt intake was more significant (*p* < 0.01) compared with that in SD rats (Figure 1).

### 2.2. Effects of High-Salt Intake on Left Ventricular Mass (LVW) and LVW Index (LVWI)

As shown in Table 1, compared with SLS group, the left ventricular mass (LVM) and left ventricular mass index (LVMI) of SHS increased significantly (*p* < 0.01), whereas no significant difference was observed in each group of SD rats.

**Table 1:** LVM and LVMI of the four groups after intervention.

Group (*n* = 8)	Body Weight (g)	LVM (mg)	LVMI (mg/g)
SLS	263.53 ± 14.36	755.11 ± 29.84	2.87 ± 0.11
SHS	290.45 ± 10.25	932.72 ± 53.97 *	3.21 ± 0.09 *
DLS	250.37 ± 23.04	749.84 ± 49.36	2.84 ± 0.11
DHS	265.83 ± 10.93	802.87 ± 59.73	2.96 ± 0.12

Abbreviations: LVM, left ventricular mass; LVMI, left ventricular mass index; SLS, salt-sensitive low salt; SHS, salt-sensitive high salt; DLS, Sprague-Dawley low salt; DHS, Sprague-Dawley low salt; * *p* < 0.01 *vs.* SLS.

### 2.3. Changes in Collagen I after High-Salt Intervention

**Figure 2:**
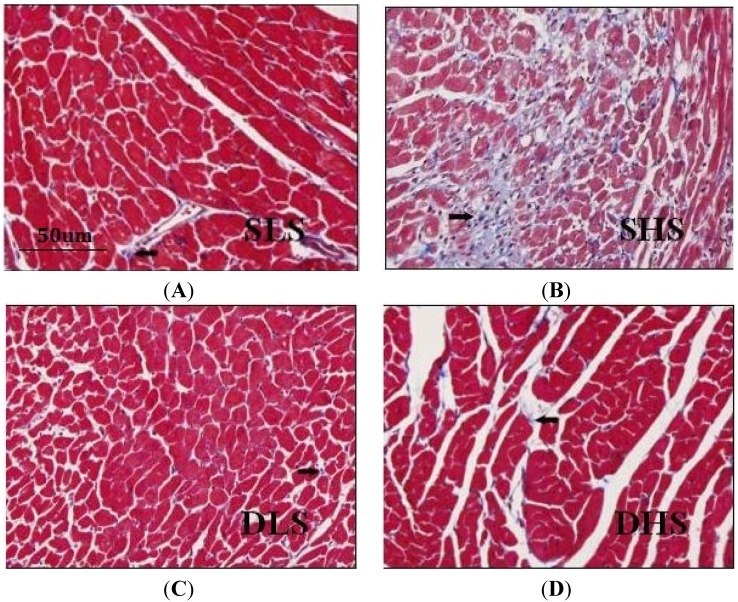
Micrographs of heart slices in the left ventricle of salt-sensitive (SS) rats and Sprague-Dawley (SD) rats. Masson’s staining was used for detection of collagen. The collagen I fibers are shown in blue. (**A**) Histologic slice of the left ventricle (LV) of SS rats received low-salt intake; (**B**) Histologic slice of the LV of SS rats received high-salt intake; (**C**) Histologic slice of the LV of SD rats that received low-salt intake; (**D**) Histologic slice of the LV of SD rats received high-salt intake. Arrows: the positive signal of collagen I fibers in myocardium.

**Figure 3:**
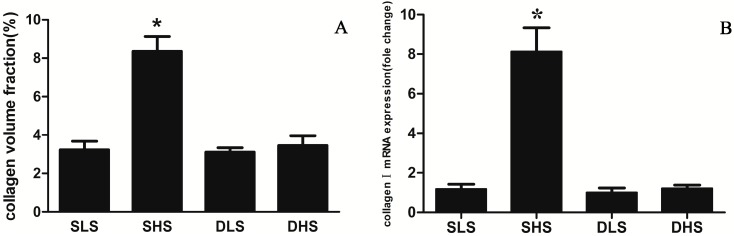
Changes in the expression of myocardial collagen I after high-salt intervention. (**A**) Changes in collagen volume fraction (CVF; %) in the four groups. * *p* < 0.01 *vs.* SLS; (**B**) Changes in mRNA for collagen I in the four groups. The mRNA expression was detected through real-time polymerase chain reaction (PCR) and normalized to the DLS group. * *p* < 0.01 *vs.* SLS.

Masson’s stain was used to show the collagen fiber that was stained blue in the myocardium. As shown in Figure 2, cardiomyocytes of the SHS rats were in disarray and collagen deposition increased significantly compared with the SLS group. The collagen volume fraction (CVF) of SHS group was significantly higher than SLS group (*p* < 0.01) (Figure 3A). By contrast, in SD rats, high-salt did not lead to collagen deposition compared with DLS group. Similarly, collagen I mRNA expression did not differ between the two strains when they were both exposed to low-salt diet (*p* > 0.05), but collagen I mRNA expression in SHS group was significantly higher than that in SLS group (8.13 ± 1.20 *vs.* 1.19 ± 0.24, *p* < 0.01). Collagen I mRNA expression was similar in DHS group and in DLS group (1.22 ± 0.17 *vs.* 1.00 ± 0.24, *p* > 0.05) (Figure 3B).

### 2.4. Effects of High-Salt Intake on Connective Tissue Growth Factor (CTGF) Expression

**Figure 4:**
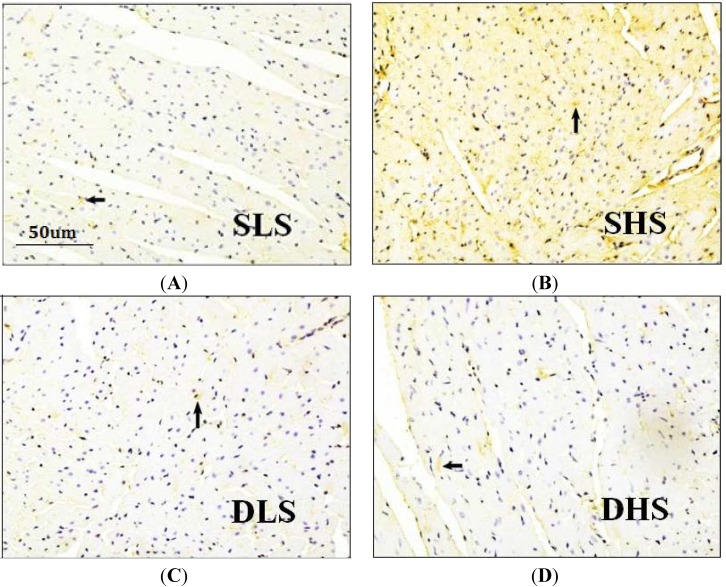
Micrographs of heart slices in the left ventricle (LV) of SS rats and SD rats. The myocardial CTGF expression was detected through immunohistochemical methods. CTGF is shown in brown. (**A**) Histologic slice of the LV of SS rats that received low-salt intake; (**B**) Histologic slice of the LV of SS rats that received high-salt intake; (**C**) Histologic slice of the LV of SD rats that received low-salt intake; (**D**) Histologic slice of the LV of SD rats received high-salt intake. Arrows: the positive signal of CTGF in myocardium.

**Figure 5:**
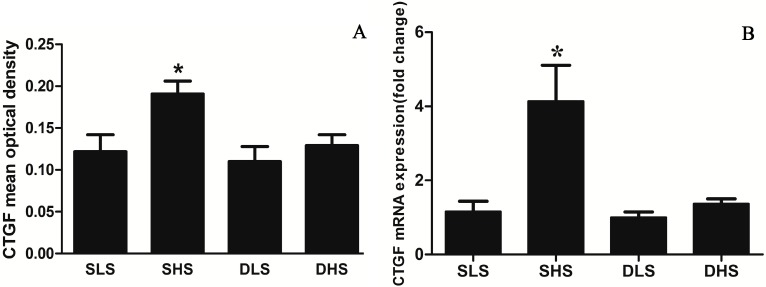
Changes in the expression of myocardial CTGF after high-salt intervention. (**A**) Changes in mean optical density (MOD)for CTGF in the four groups.* *p* < 0.01 *vs.* SLS; (**B**) Changes in mRNA for CTGF in the four groups. The mRNA expression was detected through real-time PCR and normalized to the DLS group. * *p* < 0.01 *vs.* SLS.

Immunohistochemical method was used to detect myocardial CTGF expression, and the positive signal was shown in brown. High-salt intake significantly increased the myocardial CTGF expression in SS rats, but no significant change was observed in SD rats (Figure 4). The CTGF mean optical density (MOD) in SHS group was significantly higher than SLS group (*p* < 0.01) (Figure 5A). CTGF mRNA expression in SHS group was significantly higher than that in SLS group (4.14 ± 0.97 *vs.* 1.16 ± 0.28, *p* < 0.01). No statistical difference was observed in the DHS group and the DLS group (1.37 ± 0.13 *vs.* 1.00 ± 0.15, *p* > 0.05) (Figure 5B).

### 2.5. Changes in MicroRNA-133a after the Intervention

**Figure 6:**
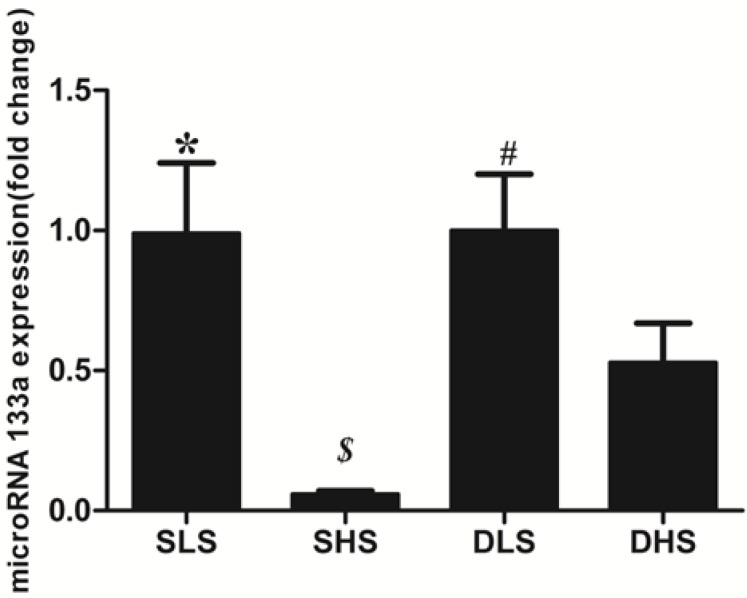
Changes in mRNA for microRNA-133a in the four groups. The mRNA expression was detected through real-time PCR and normalized to the DLS group. * *p* < 0.01 *vs.* SHS; ***^$^*** *p* < 0.05 *vs.* DHS; ^#^ *p* < 0.01 *vs.* DHS.

MicroRNA-133a was detected in the myocardial tissues of the two strains. No statistical difference was observed between the two strains when they were exposed to low-salt intake. High-salt intake decreased the expression of microRNA-133a in both strains (SS: 0.06 ± 0.01 *vs.* 0.99 ± 0.25, *p* < 0.01; SD: 0.53 ± 0.14 *vs.* 1.00 ± 0.20; *p* < 0.01). Compared with the DHS group, the decline of microRNA-133a in the SHS group was more significant (*p* < 0.05) (Figure 6).

## 3. Discussion

High-salt intake is an important environmental factor that may lead to essential hypertension. It can result in higher cardiovascular risk, myocardial hypertrophy and fibrosis, as well as vascular remodeling independently of the blood pressure [4]. Investigating the mechanism of high-salt that induces the target organ damage in hypertension would be helpful for us to understand more about the salt-sensitive hypertension.

In the current study, results indicate that compared with low-salt diet, high-salt leads to blood pressure increment in the two strains and the BP in SS rats increased more significantly. After high-salt intervention, LVM and LVMI of the SS rats were obviously higher than those of the SD rats. Other studies have confirmed that salt-sensitive hypertension entails more serious cardiac hypertrophy and fibrosis and that high salt could directly lead to cardiac fibrosis independently of blood pressure [14]. It suggests that, after high-salt intervention, the SS subjects have higher LVM and are more likely to have left ventricular hypertrophy. The onset of left ventricular hypertrophy appearing in salt-sensitive hypertension will be earlier and will be more serious than that of nonsalt-sensitive hypertension.

Studies have shown that microRNAs are involved in many kinds of pathophysiologic process and both microRNA-133a and microRNA-1 are involved in cardiac hypertrophy and fibrosis [15]. Expression of several hypertrophy-associated genes, such as calmodulin and myocyte enhancer factor 2a, sarco/endoplasmic reticulum calcium-dependent ATPase 2a and insulin growth factor 1, were negatively regulated by microRNA-1 [16,17,18]. Duisters [12] observed the downregulation of microRNA-133a in a mouse model of transverse aortic constriction and in the pathological ventricular hypertrophy of human beings. The expression of microRNA-133a is found to be negatively correlated with the expression of CTGF. Researchers have also confirmed that micorRNA-133a is bound to the 3' untranslated regions of CTGF mRNA and directly down-regulates the expression of CTGF. Using a nicotine-induced atrial fibrosis model [11], another study has demonstrated that microRNA-133a directly suppresses TGF-β expression, and that TGF-β is a upstream regulator of CTGF. Castold [13] showed that microRNA-133a could inhibit collagen I expression at the translation level by targeting three untranslated binding sites. Mass expression of collagen I is an important mark of cardiac fibrosis [19]. CTGF is a critical cytokine in the pathophysiology of fibrosis, and it could stimulate the synthesis of collagen I in myocardial cells [20]. In the current study, we found that high-salt suppresses myocardial microRNA-133a expression and increases the level of CTGF and collagen I. More studies are needed to confirm the role of microRNA-133a in salt-sensitive hypertension, and to determine whether or not it directly acts on all the three factors.

After high-salt intervention, microRNA-133a in the SD rats was suppressed by nearly 50%, but the expression of cardiac CTGF and collagen I did not increase correspondingly. For our analysis, compared with Dahl SS rats, SD rats were insensitive to high-salt intervention, a period of 4 weeks of high-salt intervention could suppress the expression of microRNA-133a, but it is not enough to cause an increase in myocardial collagen I in SD rats. Perhaps extending the high-salt intervention time will lead to an increase in the expression of CTGF and collagen I.

Activation of the local Renin–Angiolensin–Aodosterone System (RAAS), especially the concentration of *Ang*II was greatly responsible for myocardial fibrosis [21,22]. High-salt intake up-regulated the expression of AT1 receptor and elevated the *Ang*II concentration in myocardium, and this effect was independently of its effect on blood pressure [23,24]. Recently, Castold [13] showed that *Ang*II could suppress microRNA-133a expression via AT1 receptor. In the present study, we found that the expression of microRNA-133a was down-regulated both in Dahl SS rats and in SD rats, so we speculate that high-salt was likely to down-regulated microRNA expression via its effect on RAAS system in heart tissue. On the other hand, SBP of the SD rats was slightly elevated after high-salt intervention, so it indicated that high-salt intake could disturbed the expression of microRNA133a independently of its effect on blood pressure. But it is just a hypothesis and there need to be more experiments to verify it. Deleting the of AT1 receptor gene of Dahl SS rats or supplementing AT1 receptor antagonist to them may be viable methods.

## 4. Materials and Methods

### 4.1. Subjects and Protocol

Two kinds of rats were included in this study, namely, Dahl salt-sensitive (SS) rats (Charles River Company, Seattle, Washington, DC, USA) and Sprague-Dawley (SD) rats (Laboratory Animal Center, Xi’an Jiaotong University, Xi’an, China). Sixteen SS rats aged 4 weeks were randomly divided into two groups. The SS high-salt (SHS) group received 8% NaCl diet and the SS low-salt (SLS) group received 0.4% NaCl diet. Sixteen SD rats aged 4 weeks received the same treatment as the SS rats. The two groups were designated as SD high-salt (DHS) group and SD low-salt (DLS) group respectively. The intervention lasted for 4 weeks. The rats were reared in the specific pathogen free (SPF) animal room and had free access to food and water. The illumination time was 12 h/day. The institutional ethics committee of Xi’an Jiaotong University Medical School approved all experiment protocols (XJTU20120904; 4 September 2012).

### 4.2. Experimental Procedures

Blood pressure was measured at the beginning and at the end of intervention. BP2000 animal noninvasive blood pressure measuring instrument (Visitech Company, Alexandria, VA, USA) was used to determine tail systolic blood pressure. Rats received anesthesia via intra-peritoneal injection of 10% chloral hydrate. Rat hearts were acquired and the left and right atriums were cut along the atrioventricular ring. The free wall of the right ventricular was also cut along the interventricular septum. The left ventricular mass (LVW) was weighed and the left ventricular mass index (LVWI = LVW/body weight (mg/g)) was calculated. About 1/3 of the left ventricular myocardium was immersed in 4% formalin for 48 h and then embedded with paraffin, whereas 2/3 was preserved in an −80 °C refrigerator and used for RNA extraction.

### 4.3. Histological Examination of the Heart

The formalin-fixed paraffin-embedded heart tissue was stained with Masson’s trichrome (MT). For evaluation of cardiac fibrosis, the MT staining pictures were taken randomly under light microscopy (×400) from 10 different microscopic fields per section by digital camera (Nikon, Tokyo, Japan). Data are reported as the mean collagen volume fraction (CVF) in each field area. The CVF was determined by quantitative morphometry using an Image-Pro plus software (Media Cybermetics, Rockville, MD, USA).

### 4.4. Immunohistochemistry

The myocardial CTGF expression was detected through immunohistochemical methods. The antibody was from Biosynthesis Biotechnology Co., Ltd. (Lewisville, TX, USA). Immunostaining for CTGF in cardiac tissue was examined by strept avidin–biotin complex (SABC) method. Briefly, sections were deparaffinized with xylene and then dehydrated with ethanol. Endogenous peroxidase activity was inhibited for 10 min with 0.3% H_2_O_2_ in methanol, followed by a rinse with phosphate buffered saline (PBS). All sections were treated with 10% blocking solution for 30 min to prevent nonspecific antibody binding. They were then incubated overnight at 4 °C with primary antibody. After rinsing in PBS, sections were then incubated for 30 min at 37 °C with secondary antibody followed by a rinse with PBS. Then sections were treated with SP at 37 °C for 30 min. Slides were stained with DAB, counterstained in hematoxylin, dehydrated, and mounted. Control immunostaining was carried out by the same procedure in which the first antibody was replaced by PBS. Sections were photographed with a fluorescence microscopic imaging system (BX51 Olympus, Tokyo, Japan). To analyze the expression of CTGF, ten non-overlapping high-power fields (magnification 400×) were randomly selected from each cardiac section. Data are reported as the mean optical density in each field area.

### 4.5. Real-Time Polymerase Chain Reaction (PCR)

The myocardial tissue microRNA-133a, CTGF, and collagen I were detected through real-time PCR. The kits were from Takara Bio Inc. (Tokyo, Japan). GAPDH was used as internal standard for *CTGF* and *collagen* *I* mRNA detection and *U6* was used as internal standard for microRNA-133a detection. The primers used were 5'-TCGTGGAAGGGCTCATGACC-3' (sense) and 3'-TGACCTTGCCCACAGCCTTG-5' (antisense) for *GAPDH*, 5'-TGCCTACCGACTGGAAGACA-3' (sense) and 3'-TGGCAGGCACAGGTCTTGAT-5' (antisense) for *CTGF*, 5'-GGCGAAGGCAACAGTCGATT-3' (sense) and 3'-TTCCGAATTCCTGGTCTGGG-5' (antisense) for *collagen I*, 5'-ATTGGAACGATACAGAGAAGA-3' (sense) and 3'-GGAACGCTTCACGAATTTG-5' (antisense) for *U6*. The results were normalized to the DLS group.

### 4.6. Statistical Analysis

Data are presented as mean ± SD. Comparison of the blood pressure before and after the intervention was conducted through analysis of variance with the repeated measure design. Student’s *t* test was utilized to determine the difference in parameters between the rats that received different diets. Two-tailed *p* < 0.05 was considered statistically significant.

## 5. Conclusions

This study demonstrates that high-salt intake could suppress the expression of myocardial microRNA-133a partly independent of its effect on blood pressure. Myocardial down-regulation microRNA-133a may represent a regulatory mechanism that leads to myocardial fibrosis. The findings of the present study suggest that up-regulation of microRNA-133a may suppress myocardial fibrosis in salt-sensitive hypertension.
